# Association of Obesity with DNA Methylation Age Acceleration in African American Mothers from the InterGEN Study

**DOI:** 10.3390/ijms20174273

**Published:** 2019-08-31

**Authors:** Chengchen Li, Zeyuan Wang, Theresa Hardy, Yunfeng Huang, Qin Hui, Cindy A. Crusto, Michelle L. Wright, Jacquelyn Y. Taylor, Yan V. Sun

**Affiliations:** 1 Department of Epidemiology, Emory University Rollins School of Public Health, 1518 Clifton Road NE, Atlanta, GA 30322, USA; 2 New York University Rory Meyers College of Nursing, 433 First Avenue, New York, NY 10010, USA; 3 Department of Psychiatry, Yale University School of Medicine, 300 George Street, New Haven, CT 06511, USA; 4 Department of Psychology, University of Pretoria, cnr Lynnwood Road and Roper Street, Hatfield, Pretoria 0002, South Africa; 5 School of Nursing, University of Texas at Austin, 1710 Red River Street, Austin, TX 78712, USA; 6 Department of Women’s Health, Dell Medical School, University of Texas at Austin, 1701 Trinity Street, Austin, TX 78705, USA; 7 Atlanta VA Healthcare System, 1670 Clairmont Road, Decatur, GA 30033, USA; 8 Department of Biomedical Informatics, Emory University School of Medicine, 1648 Pierce Dr. NE, Atlanta, GA 30307, USA

**Keywords:** DNA methylation age acceleration, aging, obesity, BMI, African American

## Abstract

African American women are affected by earlier onset of age-associated health deteriorations and obesity disproportionally, but little is known about the mechanism linking body mass index (BMI) and biological aging among this population. DNA methylation age acceleration (DNAm AA), measuring the difference between DNA methylation age and chronological age, is a novel biomarker of the biological aging process, and predicts aging-related disease outcomes. The present study estimated cross-tissue DNA methylation age acceleration using saliva samples from 232 African American mothers. Cross-sectional regression analyses were performed to assess the association of BMI with DNAm AA. The average chronological age and DNA methylation age were 31.67 years, and 28.79 years, respectively. After adjusting for smoking, hypertension diagnosis history, and socioeconomic factors (education, marital status, household income), a 1 kg/m^2^ increase in BMI is associated with 0.14 years increment of DNAm AA (95% CI: (0.08, 0.21)). The conclusion: in African American women, high BMI is independently associated with saliva-based DNA methylation age acceleration, after adjusting for smoking, hypertension, and socioeconomic status. This finding supports that high BMI accelerates biological aging, and plays a key role in age-related disease outcomes among African American women.

## 1. Introduction

Approximately 39.8% of U.S. adults (age ≥ 20) are affected by obesity (body mass index, BMI ≥ 30 kg/m^2^) and adult obesity prevalence has increased from 30.5% to 39.6% between 2003–2004 and 2015–2016 [1]. Overall, women (41.5%) have a higher prevalence of obesity than men (38%). Among women, the prevalence of obesity is the highest among non-Hispanic African Americans (46.8%) compared with all other races and Hispanic origins [1]. Obese individuals have an increased risk for many age-related chronic diseases such as hypertension, coronary artery diseases, type 2 diabetes, and certain types of cancer [2]. Among females, non-Hispanic African Americans have the shortest life expectancy (78.1 years) compared with other racial and/or ethnic groups (Hispanic female: 84.3 years; non-Hispanic white female: 81.0 years), which could be explained by higher prevalence and earlier onset of age-related chronic conditions and comorbidity among the disadvantaged groups [3,4,5]. Data from the National Health and Nutrition Examination Survey (NHANES IV, 1999–2002) suggested that African Americans are likely to experience health deterioration earlier in their lives, and hence, age faster than whites [6].

Biomarkers that capture inter-individual variations of biological aging may accurately assess an individual’s aging-related disease risks. DNA methylation, one of the best-known epigenetic modifications, is a novel biomarker of biological aging [7]. DNA methylation modifies the biochemical property of segments of DNA to alter a gene’s expression level [8]. Recent studies have identified specific genomic regions that are subject to substantial hypermethylation or hypomethylation associated with chronological age, and demonstrated its accuracy to predict one’s age according to DNA methylation levels in specific tissues (e.g., saliva or blood), as well as across multiple tissues [7,9,10,11,12,13,14,15]. DNA methylation age (DNAm age) can be estimated based on DNA methylation levels of hundreds of CpG dinucleotides across multiple tissues [7]. Biological aging can be measured by the discrepancy between DNAm age and chronological age (Δage). Because DNAm age is linearly associated with chronological age, the residual resulting from regressing DNAm age against chronological age in a linear model can also be defined as DNA methylation age acceleration (DNAm AA). DNAm AA is independent of chronological age, and can be used as an instrument to accurately measure how fast an individual ages [16].

A limited number of studies have investigated the relationship between BMI and accelerated aging measured by DNA methylation levels among adults [17,18,19,20,21]. Only one study has been conducted among U.S. adults despite the high prevalence of obesity. Moreover, it is unclear whether findings from this study are generalizable to different age or demographic groups as well as other tissue types. In the present study, we (1) estimated DNA methylation age acceleration by applying the cross-tissue Horvath’s clock using saliva samples from 250 African American women enrolled in the Intergenerational Impact of Genetic and Psychological Factors on Blood Pressure (InterGEN) Study; (2) explored the relationship between BMI/obesity and DNA methylation age acceleration [22,23].

## 2. Results

**Figure 1:**
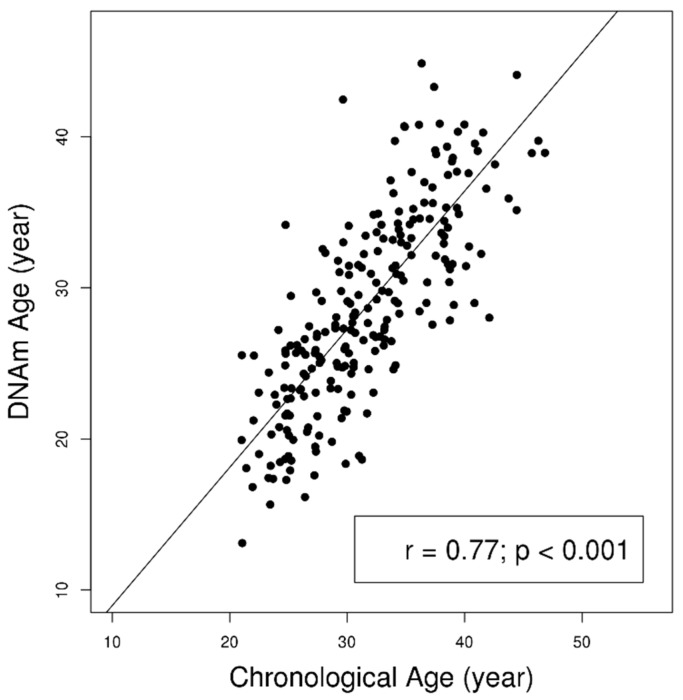
Significant correlation between the chronological age and DNA methylation age in 232. African American women from InterGEN study. The grey line indicated the fitted line from the linear regression model.

The present study included 232 African American mothers with complete measures of DNAm AA, BMI, demographic information and health history. As shown in Figure 1, chronological age was strongly correlated with DNAm Age (r = 0.77; *p* < 0.001). Table 1 summarizes sample characteristics and results of regression analysis of factors related to DNAm AA. The DNA methylation age (28.79 ± 6.75 years) was younger than the chronological age (31.67 ± 5.70 years) among our participants. The mean BMI was 29.69 kg/m^2^ (standard deviation of 8.25) with 58 overweight individuals (25.00%) and 102 obese individuals (43.97%). 53 individuals (22.84%) identified as current cigarette smokers, and 47 individuals (20.26%) reported a previous diagnosis of hypertension.

In univariate regression analysis (Table 1), we observed a statistically significant positive association between BMI and DNAm AA (0.15 years increase in DNAm AA per 1 kg/m^2^ increase in BMI (*p*-value < 0.001)). Obese individuals (BMI ≥ 30 kg/m²) were 1.87 years “older” (95% CI: 0.59, 3.16) than normal/underweight individuals. On average, overweight (25 ≤ BMI < 30 kg/m²) individuals were 1.00 years “older” (95% CI: −0.48, 2.47) than normal/underweight individuals.

However, we only observed a statistically significant difference between obese and normal weight individuals. Compared with individuals with no hypertension history, individuals with hypertension diagnosis were 1.49 years “older” measured by DNAm AA (95% CI: 0.11, 2.86). Mothers who identified themselves as current smokers were 0.73 years “younger” than non-smoker mothers, however, the association was not significant (95% CI: −2.05, 0.59). No significant associations were observed between socioeconomic factors (education level, annual household income, and marital status) and DNAm AA.

**Figure 2:**
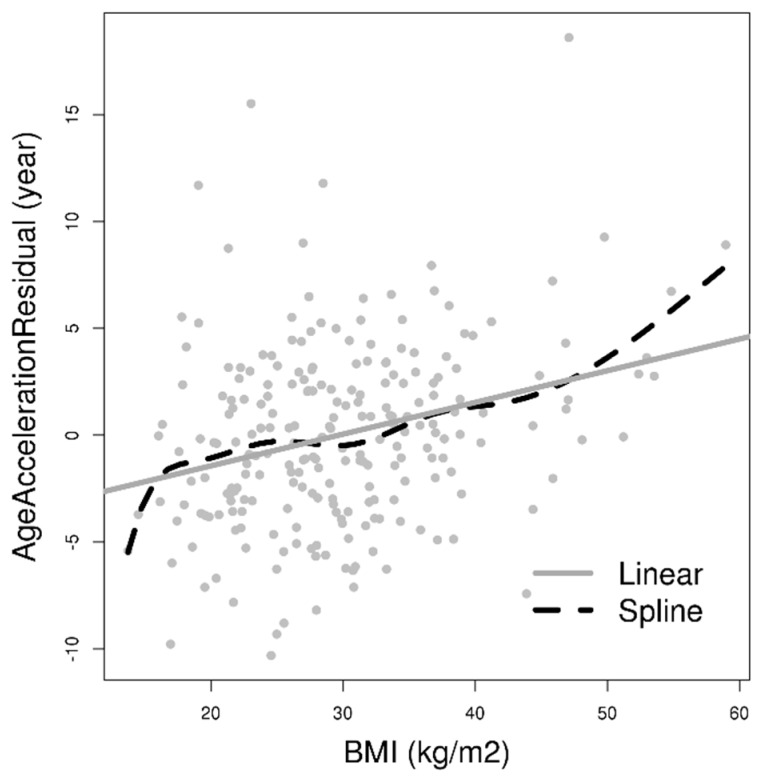
Association between BMI and DNA methylation age acceleration with a univariate linear model (0.15 years increase in DNAm AA per 1 kg/m^2^ increase in BMI (*p* < 0.001)); and a cubic smoothing spline in 232 African American women from InterGEN study. The lines indicate the fitted values from the linear and spline regression models.

To better understand the association of BMI and DNAm AA, we fit BMI as the independent variable and DNAm AA as the dependent variable in a spline regression model. We observed that DNAm AA increases more rapidly per 1 kg/m² increment of BMI among participants with low BMI (BMI < 20 kg/m²) and extremely high BMI (BMI > 40 kg/m²). This suggested that the association of BMI and DNAm AA might be stronger in individuals of low or extremely high BMI (Figure 2).

In multivariable analysis (Table 2), we further examined whether the association of BMI and DNAm AA is independent of socioeconomic status, smoking status and hypertension history. After adjusting for socioeconomic status, a 1 kg/m^2^ increase in BMI is associated with a 0.16 years increment of DNAm AA (95% CI: 0.09 to 0.22). After further adjusting for smoking status and hypertension history, BMI remained positively associated with DNAm AA. (0.14 years increase in DNAm AA per 1 kg/m^2^ increase in BMI, (95% CI: 0.08 to 0.21).

Similar analyses were conducted to investigate the relationship between weight status and DNAm AA (Table 2). As shown in Model 2 (Table 2), after adjusting for socioeconomic status, smoking status, hypertension history, and cell-type heterogeneity, obese and overweight individuals were 1.92 years (95% CI: 0.82 to 3.02) and 1.13 years (95% CI: −0.013 to 2.39) “older” than normal/underweight individuals. However, the difference was statistically significant only between obese and normal weight individuals.

**Figure 3:**
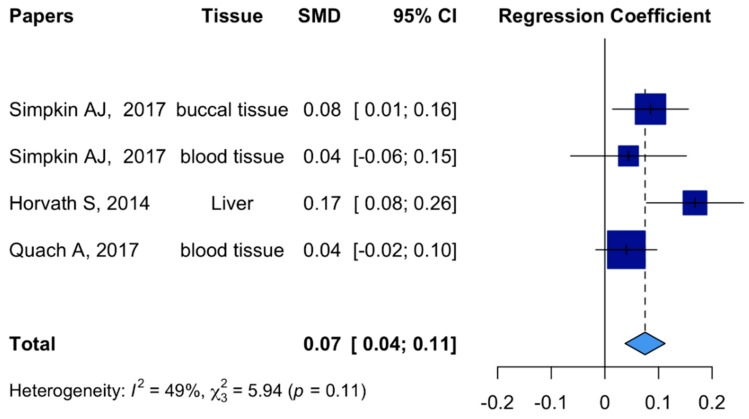
Forest plots for Horvath clock meta-analysis. Meta-analysis used regression coefficients and standard errors from three out of five studies investigating the association of BMI and Horvath’s DNA methylation aging markers. SMD: Standardized Mean Difference; Simpkin AJ 2017: Two tissues: Blood/buccal.

A systematic search for BMI association with DNAm AA was conducted in the PubMed database using terms including: adult, BMI, weight, obesity, DNA methylation age, epigenetic age, and age acceleration. Out of five relevant studies (Table S1), three studies estimated the association between continuous BMI and DNA methylation-related aging markers measured by Horvath’s clock. As shown in Figure 3, when all three studies including buccal, blood, and liver tissues were combined, BMI was positively associated with accelerated aging measured by DNA methylation. Meta-analysis indicated that 1 unit (kg/m^2^) increase in BMI was associated with 0.08 years acceleration in DNA methylation age (95% CI: 0.05 to 0.12). Due to difference in design, population, and tissue type, the associations with DNAm AA were relatively heterogeneous across studies (I^2^ = 49%).

## 3. Discussion

We reported a significant positive association between BMI and DNAm age acceleration using saliva samples from a cohort of African American mothers of at least one child aged three through five years. We also observed that this association was independent of smoking status, socioeconomic factors, and hypertension status. This positive association between BMI and accelerated epigenetic aging aligns with previous studies in different tissues and demographic groups [17,18,19,20,21]. In a subset of 4173 postmenopausal women from Women’s Health Initiative (WHI) including 2045 Caucasians, 1192 African Americans, and 717 Hispanics, a statistically significant correlation between BMI and blood-based DNAm AA among African American (r_Extrinsic DNAm AA_ = 0.05; r_Intrinsic DNAm AA_ = 0.06) was reported. Longitudinal data from the WHI also suggested that a 1 kg/m^2^ increase in BMI over time is associated with 0.22 years increase in intrinsic epigenetic AA (β_Intrinsic DNAm AA_ = 0.22), adjusted for the baseline IEAA, dataset, and ethnicity [21]. A similar association was reported in liver samples collected from a German study, where DNAm AA increased by 0.17 years per 1 kg/m^2^ increase in BMI [20]. In a British birth cohort consisting of 790 women, 1 kg/m^2^ increase in BMI was found to be positively associated with 0.085 (95% CI: 0.014 to 0.156) years increase in Δage in buccal tissue, and 0.044 (95% CI: −0.065 to 0.154) years increase in Δage for blood tissue [17]. A prospective cohort study nested within the Melbourne Collaborative Cohort collected blood samples from 2818 participants, and observed higher DNAm AAs in overweight (BMI ≥ 25) and obese individuals (BMI ≥ 30) compared with lean individuals (BMI < 25) (β_Overweight vs. Lean_ = 0.40 years; β_Obesity I vs. Lean_ = 0.15 years; β_Obesity II & III vs. Lean_ = 2.38 years) [19]. Similar to previous studies, data from the Young Finns Study indicated that BMI is positively associated with Δage measured in blood tissues in middle-age individuals (r  =  0.28). However, this study failed to observe this association among young adult and the nonagenarian populations [18]. Despite heterogeneous findings from studies with different designs, demographic characteristics, and tissue types, meta-analysis (Figure 3) of these studies suggests there is a positive cross-tissue association between BMI and DNAm AA.

Comparing results from previous reports and the meta-analysis, we observed a slightly larger estimated effect of BMI on DNAm AA in the present study (0.14 years increment in DNAm AA per 1 kg/m^2^ increase in BMI (95% CI: 0.08 to 0.21). Additionally, our findings in the correlation between BMI and DNA methylation age acceleration in saliva (r = 0.28) (Figure 2) is weaker than those reported for human liver (r = 0.42), but stronger than those for blood (0.02 ≤ r ≤ 0.08) [20,21,24]. Such tissue-dependent correlations suggest that the DNAm AA estimate may not be completely consistent across tissue types. Furthermore, unlike most of the previous studies in which most enrolled participants were older adults, our study population consists of younger African American women only [20,21]. This association and/or correlation might be dependent on age, sex, and ethnicity.

Moreover, examination of BMI categories revealed an increase of DNAm AA across higher weight groups. This trend is consistent with findings from a prospective cohort study nested within the Melbourne Collaborative Cohort, using blood samples from 2818 participants. Higher DNAm AA was observed in overweight (BMI ≥ 25) and obese individuals (BMI ≥ 30) compared with lean individuals (BMI < 25) [19].

Our observation that hypertension is significantly associated with accelerated epigenetic aging, is also consistent with other studies [25,26]. Results for self-reported smoking status and DNA methylation age acceleration are also consistent with three recent reports that did not observe any significant association [27,28,29]. We investigated the association between DNAm AA and three socioeconomic factors (i.e., annual household income, marital status, education level). Consistent with other studies, we did not observe a significant association between educational attainment and DNAm AA [30,31,32]. However, our null findings in income and marital status contrast with results from a study of a U.S. sample of middle age African American women, where significant associations were observed between low incomes, marital status, and advanced epigenetic aging estimated by Hannum’s clock [30]. InterGEN participants have limited variation of socioeconomic status, which may restrict the power to identify the association with DNAm AA.

This cohort of young African American mothers had an average ΔAge of –2.41 years and a median DNAm AA of –0.14 years (i.e., younger DNAm age than chronological age). They appear to be epigenetically “younger” despite the high burden of numerous health risk factors. It is possible that this demographic group, young African American females, were not well represented in the training of the DNAm age algorithm. Further calibration of the DNAm AA algorithm may be needed after additional validation of this finding. In fact, African Americans had lower DNAm age acceleration than Caucasians in a recent multi-ethnic study [33].

The present study investigated a novel biological aging marker, DNAm AA, among African American mothers, who tend to suffer higher prevalence of chronic disease risk factors and shorter life expectancy compared to other ethnic groups. Although this population has not developed many age-related disease outcomes, our results suggested that higher BMI and obesity may play a key role in acceleration of biological aging, even among younger age groups. Saliva samples, which consist of leukocytes and endothelial cells, have limited capability to study cell-type specific epigenetic mechanisms linked to disease etiology; however, saliva samples offer a valid and convenient DNA source for biological aging research in population studies. Our findings are based on a cross-sectional design. Thus, future studies are warranted to further understand the relationship between DNAm AA and high BMI, particularly across broader demographic groups in the context of environmental and behavioral factors. Identification of factors that increase or decrease DNAm AA will be critical for the development of interventions to reduce the impact of age-related diseases on health-related quality of life in African American women.

## 4. Materials and Methods

### 4.1. Study Sample

The InterGEN study is a longitudinal cohort study based in Southwest and Central Connecticut that examines the independent and interaction effects of genomic and environmental factors on blood pressure among African American mother-child dyads. The study procedures were reviewed and approved by New York University’s institutional Review Board (approval #1311012986, 18 April 2018). A total of 250 African American mothers and their biological children were recruited with written consent. Eligibility criteria include women who (a) speak English, (b) are 21 years of age or older, (c) self-identify as African American or Black, (d) have no active psychological or cognitive impairment that would impair their ability to reliability report on their or their children’s experiences, and (e) have a biological child aged 3–5 years. Detailed study procedures have been previously described [7,15]. In brief, members of a trained research team collect saliva samples, height and weight measurements, and blood pressure readings for both mothers and children. Demographic and socioeconomic information, psychological measures, and health history are obtained from mothers using audio self-assisted interviewing software. Researchers collect the clinical data and psychological measures during the baseline visit and three follow-ups approximately 6 months apart over the 2-year span. Data collected at baseline are included in the present study.

### 4.2. Phenotypes

BMI is calculated from height and weight as the weight (in kg) divided by the height (in m) squared. BMI category was assigned to each individual according to the World Health Organization standard: underweight (<18.5 kg/m^2^), normal weight (18.5–24.9 kg/m^2^), overweight (25.0–29.9 kg/m^2^), obese (≥30.0 kg/m^2^) [34]. Smoking was categorized as current smokers or non-current smokers. Marital status was classified into married, single, or other (divorced, separated, widowed, living with but not married to a significant other). Individuals were classified as a college graduate or no college degree according to self-report. Annual household income was grouped into <$15,000, or ≥$15,000.

### 4.3. DNA Methylation Profiling and Data Processing

Genome-wide DNA methylation was profiled in saliva samples from 250 African American women using the Illumina Infinium Methylation EPIC (850 K) BeadChip, which quantifies DNA methylation at over 850,000 CpG dinucleotides with near complete coverage of known genes. Fluorescent signals from the M (methylated) and U (unmethylated) probes were measured and used to determine DNA methylation level (β = max (M,0) /(∣U∣ + ∣M∣ + 100)) for each CpG site. Using detection *p*-value threshold of 0.001 for each CpG site, we excluded DNA methylation sites of which missing genotype call rate was above 10%. Additional quality control procedures of DNA methylation data have been described previously, including probe-level quality control and normalization [22,35]. We calculated DNAm Age for each individual using the saliva-based DNAm data and Horvath cross-tissue epigenetic clock based on age-related CpGs [16]. A total of 28,587 CpG sites from the cleaned 850 K methylation chip data were used to calculate the DNAm age, to estimate tissue type, and to predict sex by an online age calculator [7]. DNAm AA, a measure of age acceleration, is defined as the residual term of a univariate model regressing estimated DNAm Age on chronological age.

### 4.4. Statistical Analysis

We excluded individuals flagged with inconsistent prediction sex, and/or unrealistic estimated DNAm Age (|Chronological Age – DNAm Age| > 20, likely sample swapped between mother and child dyad). In addition, individuals with missing information on covariates were excluded. The final sample for the DNAm age analysis included 232 African American mothers. We conducted multiple linear regression analyses to evaluate the association between DNAm AA and BMI considering a range of covariates, which were selected based on prior studies of DNAm AA and availability in the InterGEN study [17,18,19,20,21,24,25,36]. We compared DNAm AA across BMI categories, adjusting for smoking, household income, education, marital status, hypertension diagnosis, and cell-type heterogeneity using a reference free method [37]. We also investigated the linear relationship between BMI and DNAm AA using spline regression analysis. All analyses were conducted in R studio with R version 3.4.3.

## 5. Conclusions

In African American women, high BMI and obesity are independently associated with saliva-based DNA methylation age acceleration, after adjusting for smoking, hypertension, and socioeconomic status. This finding supports that high BMI accelerates biological aging. Epigenetics can be a key molecular mechanism linking obesity and age-related disease outcomes among African American women.

## Figures and Tables

**Table 1:** Summary statistics of studied phenotypes and univariate regression on DNA methylation age acceleration (DNAm AA) from InterGEN mothers (sample size of 232).

Characteristics		Mean (SD)/Median (IQR)/N (%)	β (95% CI)
Chronological age, years		31.67 (5.70)	NA
DNA methylation age, years		28.79 (6.75)	NA
DAge, years		−2.41 (4.33)	NA
DNAm AA, years		−0.14 (−3.04–2.65)	NA
Body Mass Index, kg/m^2^		29.69 (8.25)	0.15 * (0.08, 0.21)
Maternal cigarettes use	No	179 (77.20)	(Ref)
	Yes	53 (22.80)	−0.73 (−2.05, 0.59)
Education	<College	94 (40.50)	(Ref)
	College graduate or higher	138 (59.50)	0.77 (−0.36, 1.90)
Annual household income, $	<15,000	110 (47.41)	(Ref)
	≥15000	122 (52.59)	0.19 (−0.93, 1.30)
Marital Status	Married	55 (23.70)	(Ref)
	Single	152 (65.50)	−0.28 (−1.62, 1.06)
	Others	25 (10.80)	−0.41 (−2.46, 1.64)
Ever diagnosed with hypertension	No	185 (79.70)	(Ref)
	Yes	47(20.30)	1.49 * (0.11, 2.86)
BMI category	Normal and Underweight (<24.9 kg/m²)	72 (31.00)	(Ref)
	Overweight (25–29.9 kg/m²)	58 (25.00)	1.00 (−0.48, 2.47)
	Obese (≥30 kg/m²)	102 (44.00)	1.87 * (0.59, 3.16)

Abbreviations: CI, confidence interval; BMI, body mass index; Ref: reference group; ΔAge, the discrepancy between DNA methylation age and chronological age; DNAm AA, DNA methylation age acceleration, the residual resulting from regression DNA methylation age on chronological age in a linear model. * *p*-value < 0.05.

**Table 2:** Results of cross-sectional multivariable analysis of association of BMI (kg/m^2^) or weight status, and DNAm AA (year) in study sample (*n* = 232) from InterGEN study.

Association between DNAm AA and Obesity-Related Traits		Model 1	Model 2
β coefficient (95% CI) for continuous BMI		0.15 ***	0.14 ***
		(0.09, 0.22)	(0.08, 0.21)
β coefficient (95% CI) for BMI Category	Normal and Underweight ^a^	(Ref)	(Ref)
	Overweight ^b^	1.23	1.13
		(−0.024, 2.48)	(−0.13, 2.39)
	Obese ^c^	2.01 *	1.92 *
		(0.92, 3.10)	(0.82, 3.02)
Covariates adjusted for in the model		Annual household income, Education, Marital Status, Smoking, 10 Principal Components, 8 Inferred Cell Types	Model 1 + Hypertension Status

Abbreviations: CI, confidence interval; BMI, body mass index; Ref: reference group; DNAm AA, DNA methylation age acceleration. * *p*-value < 0.05. ** Both models include covariates: Annual household income, Education, Marital Status, and Smoking. *** *p*-value < 0.001. ^a^ Normal and underweight was defined as a BMI of <24.9 kg/m². ^b^ Overweight was defined as a BMI of ≥25 kg/m² and <30 kg/m². ^c^ Obesity was defined as a BMI of ≥30 kg/m².

## Supplementary Materials

Supplementary materials can be found at https://www.mdpi.com/1422-0067/20/17/4273/s1. Table S1. Studies investigating the association of BMI and Horvath’s DNA methylation aging markers.

## Abbreviations

BMI	Body mass index
DNAm	DNA methylation
AA	Age acceleration
CI	Confidence interval
