# Supplementary material for: Association of Obesity with DNA Methylation Age Acceleration in African American Mothers from the InterGEN Study
Source: Int J Mol Sci. 2019 Aug 31;20(17):4273. doi: 10.3390/ijms20174273 (PMC6747309; doi:10.3390/ijms20174273)
Supplement: Supplementary file 1 [file ijms-20-04273-s001.pdf]

Supplementary Table S1. Studies investigating the association of BMI and Horvath's DNA methylation aging markers.

Study	Population	Sample Size	Aging Biomarker	Tissue	Type of Estimate	Effect Size	Test Statistics
Simpkin AJ, 2017	British women	790	ΔAge	buccal	β coefficient	0.085 years per kg/m2	95% CI: (0.014, 0.156)
Simpkin AJ, 2017	British women	152	ΔAge	blood	β coefficient	0.044 years per kg/m2	95% CI: (−0.065, 0.15)
Horvath S, 2014	German	141	Chronological age-adjusted DNAm Age	liver	β coefficient	0.168 year per kg/m2	SE = 0.046
Quach A, 2017	African American postmenopausal women	1058	DNAm AA (EEAA)	blood	β coefficient	0.11 years per kg/m2	p = 0.003
Quach A, 2017	African American postmenopausal women	1058	DNAm AA (IEAA)	blood	β coefficient	0.04 years per kg/m2	p = 0.17
Dugue PA, 2018	Overweight Australian adults vs. Lean adults	1384	DNAm AA	blood	β coefficient	0.40 years	SE = 0.31
Dugue PA, 2018	Obese I Australian adults vs. Lean adults	451	DNAm AA	blood	β coefficient	0.15 years	SE = 0.42
Dugue PA, 2018	Obese II Australian adults vs. Lean adults	105	DNAm AA	blood	β coefficient	2.38 years	SE = 0.73
Nevalainen T, 2017	young adults	183	DNAm Age	blood	correlation coefficient	0.11	p = 0.138
Nevalainen T, 2017	middle-aged adults	183	DNAm Age	blood	correlation coefficient	0.218	p = 0.0001
Nevalainen T, 2017	nonagenarian individuals	119	DNAm Age	blood	correlation coefficient	–0.115	p = 0.211

Abbreviations: CI, confidence interval; BMI, body mass index; ΔAge, the discrepancy between DNA methylation age and chronological age; DNAm AA, DNA methylation age acceleration, the residual resulting from regression DNA methylation age on chronological age in a linear model; β, regression coefficient from investigating the relationship between BMI and Horvath's DNA methylation aging markers; r, Spearman's rank correlation coefficient from investigating the relationship between BMI and Horvath's DNA methylation aging markers.
